# 37 Developing a practical tool for reaching consensus about shared outcome measurement in cross-sector health-enhancing physical activity partnerships

**DOI:** 10.1093/eurpub/ckae114.158

**Published:** 2024-09-26

**Authors:** Vasiliki Kolovou, Diane Crone, Nicola Bolton

**Affiliations:** Cardiff Metropolitan University, Cardiff, United Kingdom; Cardiff Metropolitan University, Cardiff, United Kingdom; Cardiff Metropolitan University, Cardiff, United Kingdom

## Abstract

**Purpose:**

To identify specific needs, priorities, and challenges to cross-sector partnerships, and to develop a practical tool for consensus-building on shared outcomes.

**Methods:**

This work included a desk-based collation of case studies (cross-sector partnerships) and a Delphi study to seek input on the tool’s design, functionality, and usability. During Round 1, a survey was launched in the HEPA conference 2023 and conference participants were invited to complete a qualitative online survey, to define key terms and definitions across sectors, and for recommending desirable partnership outcomes. Open-ended questions were used to encourage diverse perspectives and a wider range of opinions. Inductive content analysis will be used to identify and inform the development of statements for Round 2. A stakeholder workshop may be planned to engage participants and a wider audience for using and refining the partnership tool.

**Results:**

Case studies (N = 19) were identified as promoting or increasing physical activity and being driven by cross-sector partnership working. The relationships between the partners were reviewed and the process of developing and agreeing shared outcomes was collated to inform the Delphi study. Findings from the Delphi will be presented once it is completed but are anticipated to reflect the strength of agreement across the Delphi participants (N = 18, Round 1). Both case studies and Delphi surveys will lead to a partnership tool being developed to be used by real-world practitioners to develop shared partnership outcomes.

**Conclusion:**

This study is unique in its scope and aims to develop a practical tool for guiding cross-sector partners in agreeing shared outcomes that recognise impact in the whole system. Widening criteria for success in cross-sector partnerships promotes inclusivity for stakeholders, recognises the complexity in changing health behaviours and a whole systems approach to resolving physical inactivity. Establishing a shared measurement of success in partnerships can promote diverse organisations and stakeholders to contribute to tackling physical inactivity, in line with the WHO’s Global Action Plan for Physical Activity.

**Support/Funding source:**

This project was supported by the WHO Europe office for Noncommunicable diseases, as part of the lead investigator’s, Vasiliki Kolovou’s, participation in the HEPA Early Career Development Programme.

